# Can gestures speak louder than words? The effect of gestural discourse markers on discourse expectations

**DOI:** 10.1080/0163853X.2025.2499414

**Published:** 2025-05-15

**Authors:** Merel Scholman, Schuyler Laparle

**Affiliations:** a Institute for Language Sciences, Utrecht University, Utrecht, the Netherlands; b Department of Communication and Cognition, Tilburg University, Tilburg, the Netherlands

## Abstract

Much of the research on discourse marking has focused on written text, thereby not considering signals available in the multimodal domain. It is therefore an open question to what extent comprehenders rely on nonlexical discourse signals, such as gestural discourse markers. We conducted a multimodal continuation study: 48 participants were presented with videos of a speaker narrating short stories including a contrast, a list, or an exception relation. In one condition, the target discourse relation was accompanied by a gestural discourse marker; in another condition it was not. The sound was cut out at the second relational argument, and participants were asked to provide a likely continuation. The results showed that comprehenders can infer discourse meaning from gestures, but gestural signals are not as strong as lexical connectives have been found to be. These findings contribute to our understanding of how people can achieve successful comprehension in multimodal communication.

## Introduction

When people process language, they create rich mental representations of the presented information. A crucial element of these mental representations is the construction of *discourse relations*: semantic-pragmatic links such as cause-consequence and contrast, which hold between arguments (Kehler, 2002; Mann & Thompson, 1988; Sanders et al., 1992; Webber et al., 2019). To successfully construct such relations, comprehenders can make use of a variety of cues. Lexical information plays a crucial role in discourse-level processing. For example, connectives such as *nevertheless* or *because* provide readers with instructions on how to connect arguments (Britton, 1994; Gernsbacher, 1997). However, approximately 50% of relations are not marked with connectives (Webber et al., 2019), in which case people need to use “alternative” signals. Beyond lexical items, many other (linguistic and nonlinguistic) signals can correlate with a specific meaning or interpretation in discourse. These signal–meaning correlations can enrich comprehenders’ mental representation of the discourse and elicit expectations about upcoming material. However, little is known about which other types of information help the comprehender construct discourse relations between textual elements.

The majority of the research on discourse relation marking has focused on written text, but face-to-face spoken communication is fundamentally multimodal, as comprehenders can both listen to and see the speaker. The body of literature on discourse relations has thus not considered all signals available to comprehenders in typical social interactions (but see Hinnell, 2019; Hu et al., 2023; Laparle, 2022). Given the increasing attention paid toward multimodality in communication, it is important that cognitive theories of discourse take nonverbal signals into account when modeling how comprehenders construct meaning from a discourse.

**Figure 1.**
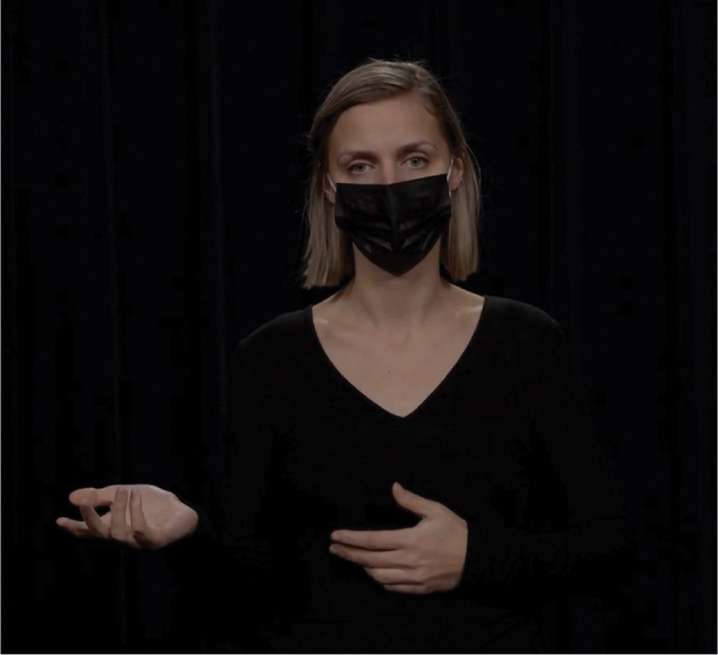
Gestural marker express contrast relations.

**Figure 2.**
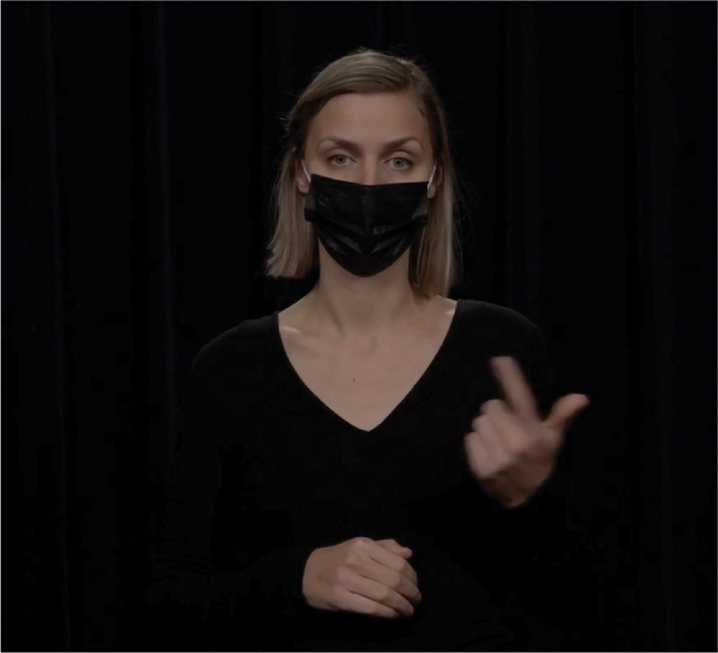
Gestural marker to express list relations.

**Figure 3.**
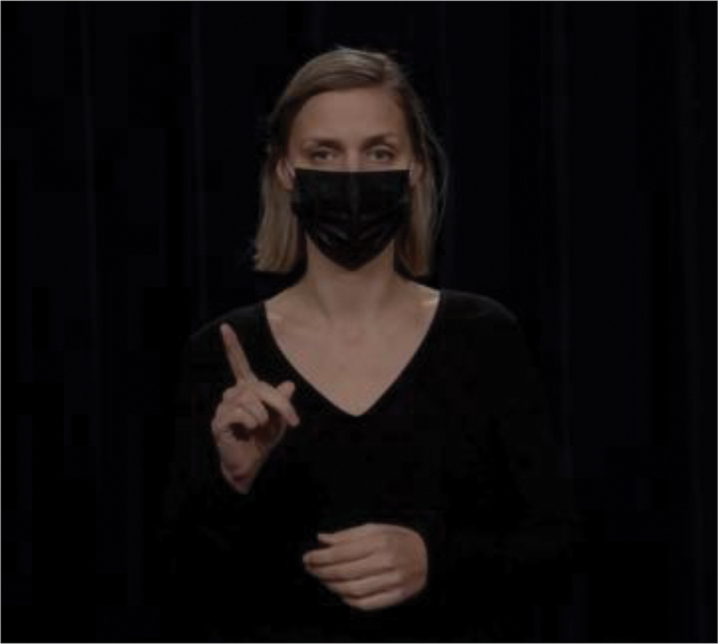
Gestural marker to express exception relations.

Hand gestures have long been known to convey pragmatic meaning similar to that conveyed by lexical markers (Bavelas, 1994; Bavelas et al., 1992; Kendon, 1995). There is also substantial evidence that speakers “design” their gestures for their addressee, depending on factors like visibility (Bavelas & Healing, 2013), bodily orientation (Özyürek, 2002), and common ground (Galati & Brennan, 2014; Höller & Wilkin, 2011). What we do not yet know is to what extent these gestural behaviors are picked up on by comprehenders for discourse processing. The current study contributes to this by investigating *to what extent comprehenders can infer discourse relational meaning from a gesture*. We focus on three types of relations for which recurrent gestures have been identified: contrast relations, which can be expressed by gesturing with a palm up open hand gesture on opposing sides of the body (Hinnell, 2019); list relations, expressed by counting with fingers (Rodrigues, 2015); and exception relations, which can be expressed using a single raised finger (indicating singularity; Inbar, 2022). These gestures are illustrated in Figures 1–3, which are stills from the videos used in the current study.

In the remainder of this section, we contextualize our research questions by reviewing previous research on discourse relational cues and on co-speech gestures. We then present a multimodal continuation study (an innovation on the monomodal continuation paradigm frequently used in discourse research), which investigates whether comprehenders can exploit gestural discourse markers to inform their discourse relation predictions.

### Discourse relational cues

Various types of discourse relational cues have been shown to influence discourse processing and representation. One of the most well-studied type of cues is connectives (e.g., *because*). Connectives have a positive influence on text comprehension (e.g., Kleijn et al., 2019; Millis & Just, 1994): Readers comprehend texts with connectives better than texts with few connectives. In addition, connectives also influence online processing: The clauses following a connective are read faster, compared to when no connective is present (Sanders & Noordman, 2000; Van Silfhout et al., 2015) or an inappropriate connective is used (Canestrelli et al., 2013; Xu et al., 2018). Moreover, material is read slower (Asr & Demberg, 2020; Canestrelli et al., 2013; Scholman et al., 2017) and yields different brain responses (Köhne-Fuetterer et al., 2021; Xiang & Kuperberg, 2015) when that material is not expected based on the connective. Such lexical cues, which are considered clear signals for discourse relations, thus influence various levels of discourse processing.

Specifically relevant to the current study is a body of work that has shown that readers use the available connectives in a discourse to inform their expectations of what discourse relation is coming next. For example, Xiang and Kuperberg (2015) found that the presence of the connective *even so* can reverse comprehenders’ expectations quickly, thereby making an unexpected event expected, that is, celebrating after failing a test (see also Köhne-Fuetterer et al., 2021). Using a similar (but monomodal) story continuation paradigm as the paradigm used in the current study, Nippold et al. (1992) found that adults provided continuations that matched the sense of 10 different connectives in 82% of the responses. Kehler et al. (2008) showed that participants always provide an explanation relation when asked to continue prompts that end with *because*. Asr and Demberg (2020) found that participants provided a continuation that matches the connective *but* or *although* in the prompts in 93% of all instances. This highlights that connectives are salient, explicit markers of discourse coherence: Their primary function is to signal discourse relations, and many connectives cannot be interpreted in other ways. They therefore have a strong influence on the way comprehenders interpret a discourse.

There are also other, nonconnective linguistic or nonlinguistic elements that tend to co-occur with a specific type of relation more than would be expected by chance. However, these cues are more implicit and ambiguous compared to connectives and therefore might not have an equally strong effect on discourse comprehension. Often, the main function of nonconnective cues is not to signal discourse relations but to convey some other meaning. For example, the negation *not* in Example 1 can be interpreted as a signal for a concession relation (which describes something being true despite an obstacle or expectation, thus going against what one might normally expect), but its primary function is to signal polarity.

(1) The cake was not entirely cooked through, but the guests still ate it.

In this example, the negation not sets up a concession by signaling that the cake is undercooked, which might suggest that it should not be eaten. However, its primary function is to negate the direct assertion that “the cake was entirely cooked through.” Negation occurs in concession relations relatively frequently (albeit together with the connective *but*; Crible, 2021). If comprehenders are sensitive to such signal∼meaning correlations, they should be able to exploit the information provided by the nonconnective signal to interpret the discourse relations. Indeed, Crible (2021) found that the presence of negation facilitated the processing of concession relations.

There is some evidence that other nonconnective lexical cues influence discourse processing. For example, Scholman et al. (2020) demonstrated that undefined expressions of quantity (e.g., *a few, several*) in the preceding context led readers to expect list relations. Participants were more likely to provide continuations that formed a list of unfortunate events when presented with a prompt like (2) compared to (3). This finding indicates that readers are attuned to nonconnective lexical cues, even across sentence boundaries, which shape their expectations about upcoming discourse relations.

(2) The woman experienced several unfortunate events last night. She got wine thrown at her by her dining companion.

(3) The woman went out for dinner last night. She got wine thrown at her by her dining companion.

However, with respect to nonlexical cues, the results are mixed. Marchal et al. (2023) found in a corpus study that gerund free adjuncts, like *painting the house* in Example (4), statistically co-occur with causal result relations in natural language (i.e., Mo was painting his house *and therefore* he was wearing old clothes).

(4) Painting the house, Mo was wearing an old sweater and ripped jeans.

A story continuation study indicated that this co-occurrence found in natural language was also reflected in readers’ offline expectations for discourse relations (Marchal et al., 2023). However, this clause structure did not facilitate the online processing or representation of results relations. In other words, readers seemed to be sensitive to the correlation between clause structure and discourse relations, but this information did not influence online processing and discourse interpretation.

Crible and Pickering (2020) studied lexical-syntactic parallelism as a signal for contrast relations, as in (5): The contrast between Nick and Grace is heightened by the repetition of the verb phrase “always eats.”

(5) Nick always eats in expensive restaurants and Grace always eats in cheap places.

In a series of self-paced reading studies, Crible and Pickering (2020) showed that contrast relations with a parallel structure were read faster than those without a parallel structure. However, this effect was only found in a task where participants were asked after reading every item whether there was a contrast in the sentence. This indicates that participants only exploited the signal when they were explicitly primed to pay attention to the relation type.

Finally, there is some evidence supporting the co-occurrence between signals in other modalities and specific discourse relations. Specifically, there is now a body of work focused on identifying systematic correlates between aspects of discourse structure and prosodic measures of pitch, pause duration, intensity and speech rate (Aubin et al., 2019; Den Ouden et al., 2009; Herman, 2000; Hirschberg & Litman, 1987, 1993; Kleinhans et al., 2017; Tyler, 2013). This line of research showed that causality is signaled by variation in pause durations and articulation rates (Den Ouden et al., 2009), that listeners can exploit the prosody on connectives to interpret causal relations as objective or subjective (Hu et al., 2023), and that prosodic correlates can be used to distinguish between coordinating versus subordinating discourse relations (Tyler, 2013). Moreover, incorporating the way different types of discourse relations tend to be realized in speech prosody leads to higher accuracy in automatic discourse relation classification systems (Kleinhans et al., 2017) and causes synthesized speech to sound more natural (Aubin et al., 2019).

Hence, it appears that discourse relations can be signaled using nonlexical auditory cues, and there is some evidence that listeners can exploit such cues (cf., Hu et al., 2023). These prosodic cues are similar to gestural cues in that they can be used independently from the choice of words or syntactic structures. Comprehenders thus have access to lexical information as well as other information from the auditory and visual modality in order to construct a coherent mental representation of the discourse. In the next subsection, we review literature on co-speech gestures and their function in discourse.

### Co-speech gestures

Gesture can be broadly construed as any “visible action when it is used as an utterance or as part of an utterance” (Kendon, 2004, p. 7). This includes the expressive hand movements that likely come to mind upon hearing the word “gesture,” but also facial expressions, head nods, shakes and tilts, and postural shifts. These visible actions are a constant accompaniment to spoken (and signed) language and can serve an array of communicative functions (see Abner et al., 2015; Mittelberg & Hinnell, 2022; Wagner et al., 2014). For example, beat gestures are rhythmic movements of the hands, driven by prosody, and serve as a physical expression of the verbal rhythm (Leonard & Cummins, 2011). While they do not carry specific meaning on their own, beat gestures can emphasize or highlight certain parts of the speech (e.g., to add emphasis to what is being said). Semantic and pragmatic gestures convey specific meaning beyond the speech itself.

In this work, we are concerned with a particular class of pragmatic gestures called *recurrent gestures* which are considered relatively conventionalized hand movements that exhibit stable form-function mappings across contexts and individuals within a speech community (Harrison & Ladewig, 2021). Recurrent gestures typically convey pragmatic information through iconicity, which is to say that some aspect of their form resembles some aspect of their meaning (Müller, 2017). Because the meaning conveyed is pragmatic, this resemblance is typically metaphoric, as the discursive objects of conversation are construed as physical objects that can be presented, pointed to, and removed. For example, the palm-up open hand gesture is a well-documented, cross-linguistically common recurrent gesture in which an open upturned hand is held toward an addressee, as if to present an object for inspection (Müller, 2004). The palm-up open hand gesture is typically associated with topic introduction, thus metaphorically mapping the topic under consideration in speech to the “object” presented on the open hand. Conversely, “flicking away” gestures, in which the hand or fingers are extended and then rapidly moved in a direction away from the body, are associated with topic dismissal and negative assessment by metaphorically mapping the topic to an unwanted object that is energetically removed from the gesturer’s immediate bodily space (Bressem & Müller, 2017). To draw a direct functional parallel to lexical markers like those discussed in the previous section, we call recurrent gestures such as these *gestural discourse markers*.

Given the iconicity of gestural discourse markers, the degree to which discourse relations are “gestureable” is likely variable; the relation must be construable metaphorically as something that can be depicted or enacted. We can partially infer the iconic potential of a discourse relation from its corresponding lexical cues. For example, a contrast relation can be expressed by the lexical marker pair *on the one hand … on the other hand* which is itself metaphoric, framing the two contrasting arguments as items placed on separate hands. This metaphoric framing of contrast can be, and indeed frequently is, iconically expressed by enacting the presentation of separated objects through two palm-up open hand gestures performed into distinct regions of space (Hinnell, 2019). Similarly, conjunction relations can be expressed by cue phrases like *furthermore* or *beyond that* which evoke a spatial metaphor locating the conjoined information in a distal region of space. One could imagine gesturally depicting this metaphoric framing by moving the hand outward. Importantly, such gestural markers can be used in the absence of the corresponding lexical cue such that, for example, contrast can be expressed iconically in gesture even when the spatial metaphor is not expressed in accompanying speech.

There is now a significant body of work on pragmatic meaning in gesture production, demonstrating recurrent mappings between gestural forms and stable pragmatic functions (see, e.g., Bavelas et al., 1992; Bressem & Müller, 2014; Bressem & Wegener, 2021; Harrison & Ladewig, 2021; Jannedy & Mendoza-Denton, 2005; Kendon, 2017; Lopez-Ozieblo, 2020; Wehling, 2017). There is also significant evidence that comprehenders pick up on the information conveyed by gesture, as the presence of gesture has been shown to increase understanding and information retention (Hostetter, 2011), increase learning outcomes in educational settings (Goldin-Meadow, 2017), and facilitate novel-task completion (Macuch Silva et al., 2020). However, we know very little about how gestural discourse markers in particular are perceived and to what extent they aid in discourse processing. The current study helps to address this by testing the capacity of gestural discourse markers to aid in discourse comprehension.

### Current study

The literature cited here shows how writers and speakers use both linguistic and nonlinguistic cues to structure discourse and signal how two clauses or sentences relate to each other. Prior studies have also shown that comprehenders exploit these cues to infer discourse relational meaning. Finally, we have discussed work that shows comprehenders perceive gestures as conveying meaningful information, but this line of research has largely overlooked the interpretation of gestural markers that can express discourse relations.

The current study asks whether comprehenders can exploit gesture to create discourse relation expectations, that is, can comprehenders infer discourse relational meaning from a gesture? In order to isolate the effect of gestures, we investigate this in the absence of verbal cues such as connectives. No studies have investigated whether comprehenders can use gestural information for inferring discourse relations. Nevertheless, we expect gestural discourse markers to present valuable information to comprehenders, based on studies showing correspondences between gesture and discourse structure.

We specifically focus on three types of discourse relations for which prior work has identified recurrent gestures. For each relation type, we study two-part gestures: the first gesture (which coincides with the first argument uttered by the speaker) sets up the frame for a certain relation; the second gesture co-occurs with the second argument and completes the discourse relation.

### Contrast

The first relation type included in the current study is the contrast relation. Logically speaking, contrast is the juxtaposing of multiple alternatives, which can be positions, arguments, options, or other propositional content (for a detailed discussion of the definition of contrast, see Molnár, 2002; Umbach, 2004). There are many different lexical and morphosyntactic ways in which contrast can be expressed, such as antonyms (*good-bad*), grammatical structure (i.e., parallelism), or prototypical connectives (e.g., *in contrast, whereas*).

**Figure 4.**
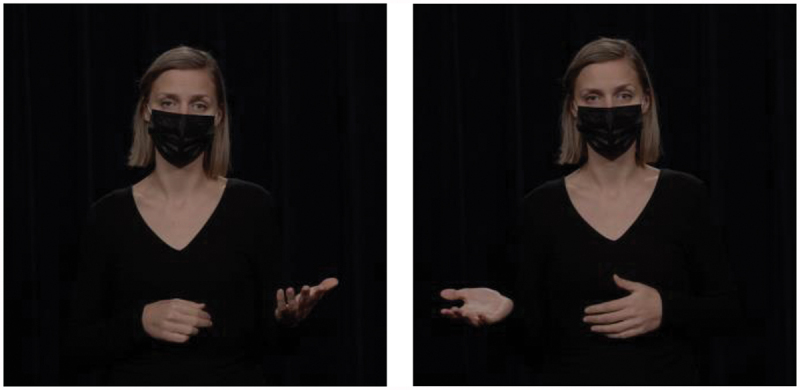
Gestural marker to express contrast relations.

The current study focuses on the embodied expression of the prototypical contrastive markers *on the one hand … on the other hand*: hands raised with their palm up and open, first on one side and then on the other side of the body, to depict the two options that the speaker is presenting. This gesture is illustrated in Figure 4. In a corpus study, Hinnell (2019) found that speakers often gesture in this way when uttering *on the one hand* (71% of instances containing this connective) and *on the other hand* (77%). This shows that the pair of markers in question is highly enacted in the body. If comprehenders are sensitive to such co-occurrence patterns, they should be able to interpret the gesture as a signal for contrast relations even in the absence of explicit contrastive lexical markers.

### List

The second relation type we consider is list. This type of relation presents multiple items, actions, or ideas in a sequential or enumerative manner. List relations can be expressed through the use of connectives such as *also* and *first … second*, or by using a grammatical structure (parallelism). Another common signal to mark list relations is prosody: The prosodic pattern is characterized by an ascending pitch at the end of the intonational unit (which coincides with the listing unit), and each new item of information tends to be introduced with a regular rhythm (Erickson, 1992).

**Figure 5.**
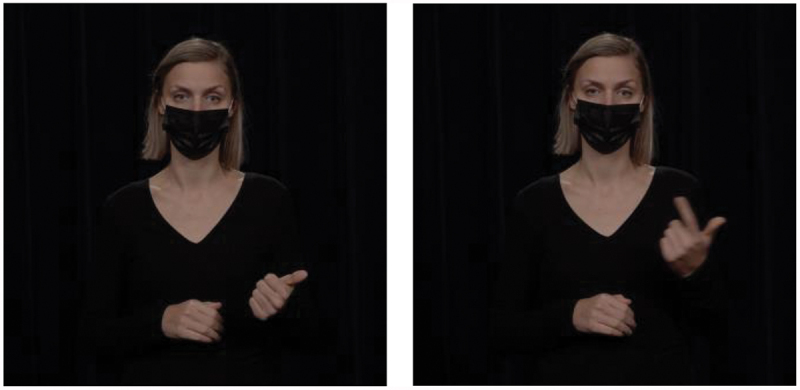
Gestural marker to express list relations.

The embodied expression of the prototypical markers *first … second* is to assign each list element to a different finger. This gesture is illustrated in Figure 5: The speaker stretched her thumb out and upward (while keeping the other fingers closed) on the first argument of the relation and additionally stretched her index finger on the same hand on the second argument of the relation. Rodrigues (2015) conducted a corpus study of gestures performed by various European and African Portuguese speakers and found that listing gestures are prevalent in many different cultures, although their form can differ slightly between cultures. For example, in Southern European cultures, the listing gestures begins with the little finger and ends with the thumb, whereas in Northern European cultures, the order is reversed. In the current study, we adhered to the Northern European style of the gesture, listing the first element on the thumb and the second element on the index finger.

### Exception

The final relation type under investigation is the exception discourse relation. This relation highlights that a particular instance deviates from the expected pattern or rule; that is, one argument introduces a general rule or generalization, and the next argument indicates one or more instances where this does not hold. This deviation from a generalization can be expressed using explicit connectives and phrases such as *except, with the exception of*, and *excluding*. More general contrastive connectives, such as *but* and *however* can also be used to express exceptions. Less explicit signals include quantifiers that refer to an almost complete set, such as *John likes most fruits*, which elicits an inference of *most but not all*.

**Figure 6.**
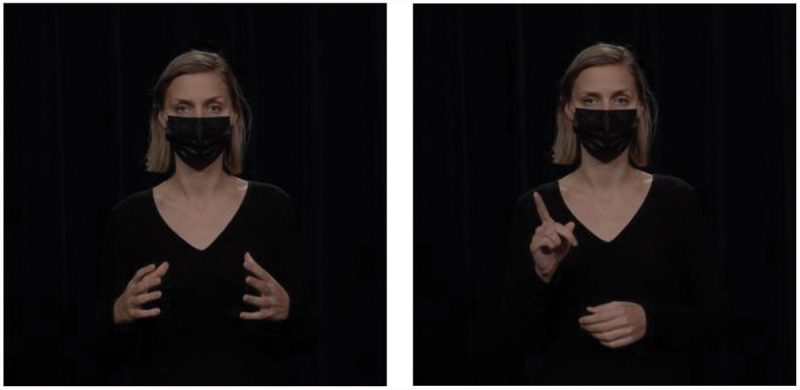
Gestural marker to express exception relations.

Hand gestures can visually represent exclusion or differentiation, as illustrated in Figure 6: The speaker first held both hands with the palms facing each other to mark a general set and then raised a single index finger to highlight the exception. The raised index finger has been described as a gesture that has a semantic core of attention (Bressem & Müller, 2014; Kendon, 2004). This gesture draws the attention of the comprehender to new and important topics of discussion. Speakers may therefore use this gesture when they assess the message to which it corresponds as unexpected, surprising, or interesting (Inbar, 2022). In a corpus study, Inbar (2022) showed that the raised index finger was most commonly used to express scalar implausibility (35% of all instances of the gesture), which relates to exception. The next most common cooccurrence of the gesture was with the opposition relation (26% of instances), which is a type of contrast relation. Note that Inbar (2022) focused only on the raised index finger, which is the second part of the exception gesture used in the current study.

We present a multimodal story continuation study, in which we investigate the role of gesture in inferring the target discourse relation that the speaker intended to convey. Participants watched videos of a speaker narrating a story. The speaker could be seen throughout the video, but the sound cut out at the second argument. Thus, participants could only see the gesture that the speaker provided, which either signaled the discourse relation (the gestural discourse marker condition) or not (the beat gesture condition). Participants were asked to write what they think the speaker said during the time the sound was cut out.

We are interested in a main effect of gesture type: we expect that comprehenders can infer discourse meaning from gestures, in which case we should see more completions matching the target relation that is expressed by the gesture in the gestural discourse marker condition than in the beat gesture condition. However, if they cannot infer meaning from the gestures, we should see no difference between the two conditions for each target relation.

Note that we do not assume that all gestural signals have an equally strong effect on discourse interpretations: both the contrast and the list gestures are more iconic and unambiguous than the exception gesture. A single raised finger does not iconically map on to the lexical signal “except” but rather expresses singularity. Inbar (2022) showed that this gesture also co-occurs with other relation types, such as concession and instantiation (a type of relation in which one argument presents an example of the other argument). We therefore expect an interaction effect between relation type and gesture type: the effect of gestural discourse marker presence on discourse interpretations should be stronger for contrast and list relations, compared to exception relations.

## Multimodal continuation experiment

The goal of this study was to determine whether comprehenders can infer meaning from gestural discourse markers in communication. The study and the analysis plan were preregistered; the preregistration and all materials, data and code are available in an online repository (https://osf.io/68mcf/).

### Methodology

#### Participants

A total of 48 native speakers of English (age range, 18–74 years; mean, 42 years; 31 women) were recruited via Prolific and received 3.50 GBP for their participation (average study duration, 20 minutes). Data from two additional participants were not included in the analysis because they did not provide usable responses: They repeated the prompt, rather than continuing it.

#### Materials

This experiment was designed as a 3 (between-item relation type: contrast, list and exception) × 2 (within-item gesture type: discourse marker or beat gesture) study. The experimental stimuli consisted of eighteen videos of a speaker narrating short stories. Each story comprised two introductory sentences as well as the first and second arguments of the target relation (arg1 and arg2, respectively). Three types of target relations were included: contrast (an example is provided in passage 6), list (passage 7) and exception (passage 8). We included six items per relation type; the written version of the stimuli is included in Appendix.

(6) *Contrast item*:

Heather loves to travel solo, and has done so multiple times. She is considering what activities to do while on vacation in Hawaii next month.*[Intro]*

She really loves the idea of learning how to surf.*[Arg1]*

On the other hand, relaxing at the beach with a nice cocktail sounds good too.*[Arg2]*

(7) *List item*:

Nathaniel has a crush on his classmate Suzy and wants to take her to prom.

He told me he has an awesome proposal planned.*[Intro]*

He practiced a choreographed dance to an Ed Sheeran song.*[Arg1]*

He also made a banner that read “Will you be my boo?.”*[Arg2]*

(8) *Exception item*:

My friend Melissa has been a big Quentin Tarantino fan since she was young.

She has posters of his films plastered up on her walls.*[Intro]*

She has basically seen all Tarantino films that exist.*[Arg1]*

Except for “Jackie Brown,” which she has not had a chance to watch yet.*[Arg2]*

Every item was recorded as a video with audio. The speaker could be seen throughout every video, but the sound was cut out in the second argument. Instead, participants heard white noise. The video continued to play, and therefore the participants could see the gesture that the speaker provided.

Prior to recording the videos, the audio was recorded separately in the phonetics lab at the Institute for Language Sciences labs, using the open-source digital audio editor and recording application software Audacity (https://www.audacityteam.org). The audio was recorded separately from the video because for every item, two videos were recorded: one with a gestural discourse marker and one with only beat gestures. We overlayed the same audio on both videos to ensure that any difference between the conditions could not be attributed to prosodic cues. The voice actress (the second author of this article) is a native speaker of American English. For all items, the entire item including arg2 was recorded. The arg2 was later removed from the audio and replaced with white noise using the software Praat.

The videos were recorded by an audiovisual engineer and a research assistant at a studio affiliated with the Institute for Language Sciences labs at Utrecht University. One actress (the first author of this article) was used as the actress for all items. The speaker wore a face mask in all videos to ensure that the participants could not read the speaker’s lips and so that a single audio file could be used in both conditions for every item.

The authors agreed on a gesture form for every relation type included (e.g., which fingers to use for the listing gesture). Training consisted of practicing every item once before recording it. Two videos were created for each item: one video in which the discourse relation was gestured (the gestural discourse marker condition), and one in which the discourse relation was not gestured through a gestural discourse marker but the speaker provided a simple beat gesture (the beat gesture condition).

The experimental items were interspersed with six filler items containing similar story structure; three of these contained a temporal gesture in which the hand moved as if to toss something back over the shoulder. These items were intended for a different study and were not analyzed as part of this study.

### Norming studies

Materials were normed in three norming studies. We first conducted a written story continuation study (*n* = 20) and an auditory story continuation study (*n* = 20) to eliminate items that were inherently biased toward the target relation and thus left little room for the gesture to steer interpretations. For these two norming studies, participants were presented with the context and first argument in a written modality or an audio modality (without viewing the video), and they were asked to continue the story. These continuations were annotated to determine whether they matched the target relation or not. All items included in the main experiment did not receive any target continuations in these two norming studies, thereby limiting the probability of lexical and prosodic cues signaling the target relation and leading to a possible ceiling effect. Consequently, any effect that might be found in this experiment can be attributed to the gesture rather than other sources of information.

We conducted a third norming to confirm the acceptability of the gestures that were identified in the literature for the types of stories used in this study. Fifteen participants were asked to watch a subset of the experimental videos and rate on a scale of 1 to 5 if they thought the gesturing was “natural” and “appropriate.” Four instances of each relation type were included in this norming study (contrast, list, and exception); two in each gesture type condition (gestural discourse marker condition and beat gesture condition). The items were presented with the full audio, including arg2 which contained an explicit lexical connective. The items were interspersed with three fillers containing exaggerated gestures not related to what was being said (e.g., waving both hands in front of the face) to create a range of naturalness. The norming study also contained four videos with a temporal relation and corresponding gesture (for an unrelated study).

All results reported in this paper were modeled using R and Rstudio (R version 4.3.2, RStudio Team, 2020). We analyzed the naturalness and appropriateness ratings separately using ordinal mixed-effects regression models, implemented via the cumulative link mixed model (CLMM) framework. CLMMs are specifically designed to model ordinal response data, such as Likert-scale ratings, without assuming that the intervals between rating points are equal. Instead of treating the response as continuous, CLMMs estimate the probability of a response falling into or below a given category, based on an underlying latent continuous variable. This approach preserves the ordinal nature of the data and avoids the distortions that can result from treating ordinal outcomes as interval-scaled, making CLMMs more appropriate than linear models for this type of analysis (Taylor et al., 2023).

The models were created with the ordinal::clmm function and evaluated using the lme4 package. Models were fit with a logit link and flexible thresholds to account for the possibility that participants did not treat the distances between adjacent Likert-scale points as psychologically equal. We included centered Likert ratings as the response variable and maximal random effect structures.

*Norming study: Comparison of experimental and filler items*. The first analysis was designed to test whether the experimental items were considered relatively natural compared to the three exaggerated filler items. The model contained fixed effects for item type (experimental vs. filler, sum-coded) and random intercepts for both item and subject. Additionally, the model included by-item and by-subject random slopes for item type. For the appropriateness model, the by-item random slope was removed for convergence.

Table 1 shows the mean ratings for filler items and experimental items. Tables 2 and 3 show that experimental items received higher ratings than exaggerated filler items for both naturalness and appropriateness. This indicates that the experimental items were considered (relatively) natural by the participants. Note that the full random effects model for this data did not yield standard errors due to an undefined variance-covariance matrix, likely caused by overfitting because filler items always tended to be rated low. We therefore simplified the model by reducing the random slope structure to achieve stable estimates.

**Table 1.** Mean raw and latent ratings for the naturalness and appropriateness of items in exaggerated fillers and experimental items, norming study.

	Naturalness rating		Appropriateness rating	
Item type	Raw *M* (*SD*)	Latent *M* (*SE*)	Raw *M* (*SD*)	Latent *M* (*SE*)
Filler	1.87 (1.04)	−2.73 (0.66)	1.87 (1.06)	−2.71 (0.84)
Experimental	3.72 (1.06)	1.56 (0.42)	3.96 (1.02)	1.99 (0.41)

**Table 2.** Regression coefficients and test statistics for the difference between exaggerated fillers and experimental items from the ordinal mixed-effects model for naturalness ratings; norming study.

Threshold coefficients	β	*SE*	*z*	*p*
−2.35|-1.35	−2.38	0.44	5.45	*<*.**001**
−1.35|-0.35	−0.40	0.40	−0.99	.32
−0.35|0.64	1.64	0.41	3.98	*<*.**001**
0.64|1.64	3.49	0.45	7.78	*<*.**001**
**Fixed effect**				
Item type	2.14	0.41	5.14	*<*.**001**
**Random effects**	Variance	*SD*		
Subjects – Intercept	0.36	0.60		
Subjects – Item type	0.31	0.55		
Items – Intercept	0.80	0.89		
Items – Item type	1.07	1.04		
**Model summary**				
ICC	0.39			
N items	15			
N subjects	15			
Observations	225			
Marg. R^2/^Cond. R^2^	0.353/0.607			

Model formula: rating ∼ item type + (1+ item type|item) + (1+ item type|subject).

**Table 3.** Regression coefficients and test statistics for the difference between exaggerated fillers and experimental items from the ordinal mixed-effects model for appropriateness ratings; norming study.

Threshold coefficients	β	*SE*	*z*	*p*
−2.53|-1.53	−2.24	0.52	−4.33	*<*.**001**
−1.53|-0.53	−0.84	0.49	−1.70	.09
−0.53|0.46	1.40	0.50	2.81	*<*.**001**
0.46|1.46	3.12	0.52	5.97	*<*.**001**
**Fixed effect**				
Item type	2.35	0.48	4.92	*<*.**001**
**Random effects**	Variance	SD		
Subjects – Intercept	1.05	1.03		
Subjects – Item type	0.68	0.82		
Items – Intercept	0.83	0.91		
Subjects – Intercept	1.05	1.03		
**Model summary**				
ICC	0.37			
N items	15			
N subjects	15			
Observations	225			
Marg. R^2/^Cond. R^2^	0.403/0.626			

Model formula: rating ∼ item type + (1|item) +

(1+ item type|subject). The random effects structure for item was simplified to achieve stable estimates.

*Norming study: Comparisons across gesture and relation types*. The second analysis was designed to test the effect of gesture type (gestural DM or beat gesture) and relation type (contrast, list and exception). The fillers were excluded from this analysis. The model contained fixed effects for gesture type condition (sum-coded) and relation type condition (deviation-coded with exception as reference level) and their interaction, as well as random intercepts for item and subject. Additionally, it included a by-item random slope for gesture and by-subject random slopes for gesture, relation, and their interaction.

Table 4 presents the mean ratings for naturalness per gesture type and relation type condition, and Table 5 present the test statistics for the model comparing naturalness ratings for the target relations. Tables 6 and 7 presents the same for the appropriateness question. The results show a main effect of gesture condition for both naturalness and appropriateness: videos with a gestural discourse marker were rated as more natural and appropriate than videos with only beat gestures. No difference was found between relation types, nor was there an interaction effect between gesture presence and relation type. Thus, the results indicate that the included gestures were appropriate to express the target relation types, and that including them made communication seem more natural.

**Table 4.** Mean raw and latent naturalness ratings for items in the three target relation conditions, per gesture condition.

Target relation	Gestural DM		Beat gesture	
	Raw *M* (*SD*)	Latent *M* (*SE*)	Raw *M* (*SD*)	Latent *M* (*SE*)
Contrast	4.33 (0.92)	3.85 (0.62)	3.03 (1.19)	0.24 (0.82)
List	4.17 (0.83)	3.29 (0.60)	3.50 (0.78)	1.25 (0.50)
Exception	4.17 (0.79)	3.14 (0.49)	3.13 (1.04)	0.38 (0.66)

**Table 5.** Regression coefficients and test statistics from the ordinal mixed-effects model for naturalness ratings; norming study.

Threshold coefficients	β	*SE*	*z*	*p*
−2.72|-1.72	−5.64	0.74	−7.58	*<*.**001**
−1.72|-0.72	−3.62	0.52	−6.94	*<*.**001**
−0.72|0.27	−0.59	0.38	−1.57	.12
0.27|1.27	1.75	0.39	4.47	*<*.**001**
**Fixed effect**				
Gesture type	1.40	0.32	4.41	*<*.**001**
List	0.51	0.46	1.10	.27
Contrast	0.28	0.45	0.63	.53
Gesture type:list	−0.36	0.40	−0.91	.36
Gesture type:contr.	0.42	0.44	0.97	.33
**Random effects**	Variance	*SD*		
Subjects – Intercept	1.33	1.15		
Subjects – Gesture type	1.03	1.01		
Subjects – List	1.03	1.01		
Subjects – Contrast	0.87	0.93		
Subjects – Gesture type:list	0.28	0.53		
Subjects – Gesture type:contr.	0.73	0.85		
Items – Intercept	0.00	0.00		
Items – Gesture type	0.00	0.00		
**Model summary**				
ICC	0.39			
N items	12			
N subjects	15			
Observations	180			
Marg. R^2^	0.392			

Model formula: rating ∼ gesture * relation +

(1+ gesture|item) + (1+ gesture*relation|subject).

The reference level is the exception target relation.

Conditional R^2^ was not reported due to near-zero item variance.

**Table 6.** Mean raw and latent appropriateness ratings for items in the three target relation conditions, per gesture condition.

Target relation	Gestural DM		Beat gesture	
	Raw *M* (*SD*)	Latent *M* (*SE*)	Raw *M* (*SD*)	Latent *M* (*SE*)
Contrast	4.43 (0.90)	4.17 (0.66)	3.27 (1.11)	0.73 (0.63)
List	4.47 (0.63)	4.03 (0.62)	3.80 (0.82)	1.97 (0.54)
Exception	4.43 (0.68)	3.88 (0.59)	3.33 (1.09)	0.79 (0.65)

**Table 7.** Regression coefficients and test statistics from the ordinal mixed-effects model for appropriateness ratings; norming study.

Threshold coefficients	*β*	*SE*	*z*	*p*
−2.95|-1.95	−5.97	0.76	−7.83	*<*.**001**
−1.95|-0.95	−4.23	0.55	−7.73	*<*.**001**
−0.95|0.04	−1.23	0.35	−3.51	*<*.**001**
0.04|1.04	1.05	0.33	3.16	*<*.**001**
**Fixed effect**				
Gesture type	1.43	0.31	4.55	*<*.**001**
List	0.67	0.44	1.50	.13
Contrast	0.12	0.54	0.21	.83
Gesture type:list	−0.51	0.38	−1.35	.18
Gesture type:contr.	0.18	0.39	0.46	.64
**Random effects**	Variance	*SD*		
Subjects – Intercept	1.69	1.30		
Subjects – Gesture type	0.87	0.93		
Subjects – List	0.79	0.89		
Subjects – Contrast	2.14	1.46		
Subjects – Gesture type:list	0.68	0.82		
Subjects – Gesture type:contr.	0.68	0.82		
Items – Intercept	0.00	0.00		
Items – Gesture type	0.00	0.00		
**Model summary**				
ICC	0.37			
N items	12			
N subjects	15			
Observations	180			
Marg. R^2^	0.404			

Model formula: rating ∼ gesture * relation +

(1+ gesture|item) + (1+ gesture+relation|subject).

The reference level is the exception target relation.

Conditional R^2^ was not reported due to near-zero item variance.

### Procedure

The story continuation study was implemented on Qualtrics. The stimuli (written version of the materials shown in Appendix A; the videos are available on OSF) were counterbalanced across two lists, with each story appearing in a different gesture condition but the same relation condition in each list. All participants saw each story in only one condition. The participants were randomly assigned to one of the lists, and for each participant the list was presented in a unique order. List 1 was completed by 27 participants; list 2 by 23 participants. Participants were asked to watch each video and write a continuation of what the speaker might have said while they heard white noise.

The continuations were coded by one of the authors to determine whether the participants constructed a relation that matched the gestural discourse marker (i.e., contrast continuations in the contrast condition; list continuations in the list condition; exception continuations in the exception condition). The coding rubric is available on OSF. When a participant provided a continuation that corresponded to the focal relations of contrast, list, and exception in a different condition, such as a contrast continuation in the list condition, it was annotated as a nontarget continuation.

Passage (9) presents some examples of the different types of continuations provided by the participants.

(9) *List prompt*: Ruben is very concerned for the environment. At a family event, he was explaining to his uncle how the environment is in grave danger. The sea ice is melting at a shockingly rapid pace.

- *Target list continuation*: Ruben also told his uncle that CO_2_ from industrial activities all over the world was accumulating rapidly in the upper atmosphere. Ruben also said that electric or solar powered vehicles may be needed to minimize the threat.
- *Nontarget continuation (temporal*): Just as Ruben was completing his statement about the environment’s health, he was rudely interrupted.
- *Nontarget continuation (causal*): He decided he would join an environmental group. And eventually became the leader of that group. And made a big difference in global warming.
- *Nontarget continuation (concession*): But Ruben is worried of the next action to take and then called his senior brother.
- *Nontarget continuation (expansion*): He is so passionate about his beliefs he talked to anyone that would listen.

One observation was missing due to a technical issue. This left us with a set of 899 continuations. Of these continuations, a random subset of 10% was double-coded by a second, experienced coder. Annotators were blind to condition. We calculated Cohen’s κ to evaluate interannotator agreement (Cohen, 1960; see Landis & Koch, 1977; Spooren & Degand, 2010 for a discussion of what constitutes sufficient interannotator agreement). For the current study, the agreement between the coders was almost perfect: 93%, Cohen’s κ = .86. Thirty-nine observations (36 of which belonged to two participants) were not usable continuations; they merely repeated the prompt. These observations were removed, leaving us with 860 observations that were included in the analysis.

We constructed generalized mixed-effect regression models, with a binary variable for target response (1 = matching target relation) as response variable and fixed effects for gesture type condition (deviation-coded), relation type condition (simple-coded with exception as reference level) and their interaction. We initially sought to specify the maximal random effect structure for our model, which included random intercepts and random slopes for both subjects (i.e., allowing for varying sensitivities to the gesture condition and relation type condition across participants) and items (i.e., allowing for varying sensitivities to the gesture condition across items). However, during model fitting, we encountered singularity issues, which typically arise when the random effects structure is too complex, making it difficult for the model to converge. To address this, we tested different combinations of random effects, systematically removing random slopes to identify the most complex model that would still converge. Ultimately, we found that none of the alternative combinations (e.g., random slopes for gesture condition or relation type condition across participants or items) led to a converging model. As a result, we simplified the random effects structure by removing all random slopes, leaving only random intercepts for both subjects and items.

### Results

**Figure 7.**
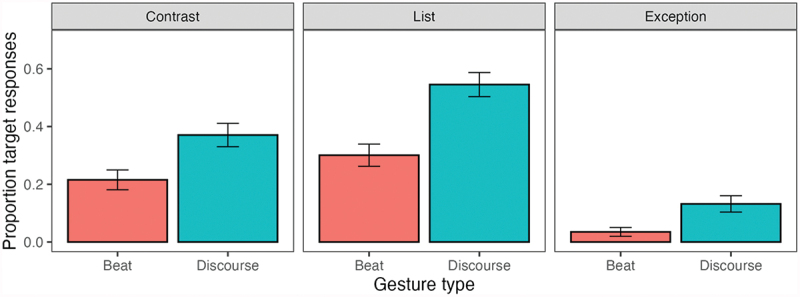
Proportion of target continuations (and standard error bars) per condition and relation type.

Figure 7 displays the proportion of target responses per relation type and gesture condition; the model results are shown in Table 8. The random slopes for item and subject were removed due to singularity issues. The model showed a main effect of gesture condition: videos with a gestural discourse marker received a higher proportion of target continuations than videos with only beat gestures. Items in the contrast and list target conditions received more target continuations than exception items. Crucially, there was no interaction between condition and target relation, indicating that the gestural discourse markers led to a significantly higher proportion of target continuations in all three relation types.

**Table 8.** Regression coefficients and test statistics from a generalized mixed-effects model for the effect of gesture type and relation type conditions.

Fixed effects	*β*	*SE*	*z*	*p*
(Intercept)	−1.78	0.40	−4.42	
Gesture type	0.69	0.12	5.64	*<*.**01**
List	2.99	0.92	3.26	*<*.**001**
Contrast	2.21	0.91	2.42	.**02**
Gesture type:list	0.21	0.33	0.64	.53
Gesture type:contrast	−0.13	0.32	−0.39	.69
**Random effects**	Variance	*SD*		
Subjects	Intercept	1.15	1.07	
Items	Intercept	2.01	1.42	
**Model summary**				
ICC	0.49			
N items	18			
N subjects	48			
Observations	860			
Marg. R^2^ /Cond. R^2 ^=	0.246/0.616			

Model formula: response ∼ gesture * relation +

(1|item) +(1|subject).

The reference level is the exception target relation.

**Figure 8.**
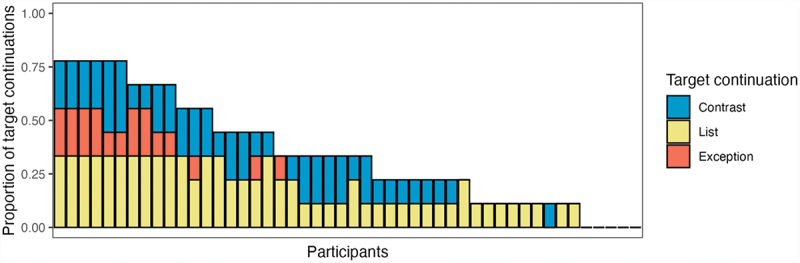
Mean proportion of target continuations per participant and target relation type. Every bar represents one participant.

We also consider the proportion of target continuations provided per participant in the gestural discourse marker condition, to determine whether participants varied from each other in their sensitivity to the gestural discourse markers. The mean percentage of target continuations provided was 34%. The frequency of target continuations per participant ranged between 0% and 78%. This is visualized in Figure 8, which displays the proportion of target continuations for every participant. Note that a proportion of 1 indicates that the participant provided the target continuation to all 9 items in the gestural discourse marker condition (3 per relation type). In total, 5 of 48 participants did not provide any target continuations in the gestural discourse marker condition, indicating that they did not respond as predicted to any of the three gestural discourse markers in any of the items at all.

## Discussion

The current study set out to investigate to what extent comprehenders can infer discourse relational meaning from gestures. We focused on three types of relations for which recurrent gestures have been identified: contrast, list, and exception relations. We conducted a multimodal story continuation study, which showed that comprehenders do take into account the provided gesture when inferring what a speaker might have said: participants were more likely to provide continuations that matched the target relations in the presence of a gestural discourse marker. This study therefore provides evidence that gestures can be used to express abstract discourse structural information.

However, the gestural discourse markers included in the current study did not influence discourse interpretations as strongly as certain lexical connectives have been found to. As discussed in the Introduction, various studies have shown that readers use the available connectives to inform their expectations of what discourse relation is coming next. For example, Kehler et al. (2008) found that participants always provide an explanation relation when asked to continue a prompt with *because* (a clear, unambiguous connective), and Asr and Demberg (2020) found that participants provided a continuation that matched the connectives in the prompts (specifically, *but* and *although*) in 93% of all continuations. Compared to those results, the current study showed relatively low rates of target continuations: 37% for the contrast gesture, 55% for the list gesture, and 13% for the exception gesture.

The contrast gesture was less effective than an explicit contrastive connective. Scholman et al. (2017) used prompts similar to those in the contrast condition of this study and found that participants provided a contrast continuation for 75% of stories containing “On the one hand” (presented without a second contrastive element or the marker “on the other hand”). In contrast, the list gesture was *more* effective in guiding comprehenders’ interpretations compared to an alternative lexical signal: Scholman et al. (2020) found that in a story continuation paradigm, 35% of prompts containing a quantifier like *several* were followed by a list, compared to our 55%. Thus, the gestural discourse marker had a stronger influence on discourse expectations than a lexical nonconnective signal. Future research could examine the relative strength of various gestures compared to other signals. It is likely that, similar to gestural discourse markers, not all connectives or nonconnective signals exert an equally strong influence on discourse expectations. Understanding these differences will provide insight into how comprehenders infer meaning in discourse, contributing to more effective communication strategies.

Regarding the exception gesture, we had expected this gesture to result in a lower proportion of target continuations than the other two gestures due to its observed polysemy in corpus work (Inbar, 2022). The results showed an effect that appeared close to floor level. This could be due to the relation type or the gesture used to express exceptions. First, exception relations are less common in natural language compared to other types of relations such as causal relations, and participants might not even have considered the target relation. Second, participants might have interpreted the gesture in a different way, such as indicating the numeral “1.” Indeed, 11% of continuations offered for exception items in the gestural discourse marker condition included the word ”one”, but were not exception relations (e.g., prompt: *My friend Melissa has been a big Quentin Tarantino fan since she was 15. She has posters of his films plastered up on her walls. She has basically seen all Tarantino films that exist*. Continuation: *One day she would love to meet him*.). Thus, exception continuations might have been low because the single raised finger is an ambiguous signal with a competing semantic meaning. Future work can focus more specifically on the issue of competing semantic and pragmatic interpretations of a single raised finger.

There are several possible explanations for the difference in relative strength between connectives (as found in earlier studies) and gestures (as found in the current study). First, signal transparency might influence relative strength: Gestures can be meaningful but can also be unintentional hand movements or be used in relation to something in the environment (and not necessarily the communication). In comparison, lexical connectives might be clearer, more intentional, and less ambiguous signals than gestural markers. Comprehenders might therefore assign less prominence to gestural signals than to lexical signals. This could be tested in a paradigm in which participants are explicitly prompted to pay close attention to the gesture to infer the intended meaning. If participants show higher rates of target continuations, this might indicate that signal prominence plays a role in the extent to which comprehenders derive discourse relational meaning from gestural markers. A second possibility is that familiarity with the signal might influence relative strength: comprehenders might have had more exposure to the lexical signal (in both spoken and written language) compared to the gestural signal (only in visual settings). If this is indeed true, we would expect that comprehenders who have a higher degree of print exposure would be more sensitive to (nonconnective) lexical signals, but not to visual signals. Such a crossmodal comparison could provide interesting results related to the effect of contextual factors on comprehension.

Although the current study indicates that the relative strength of gestural discourse markers might be less than that of explicit lexical connectives, the results presented here do provide evidence that gestural discourse markers can influence comprehension. These results have an important implication for theories of discourse comprehension. Traditionally, the field’s focus has been on the semantic encoding of discourse relations. However, a purely connective-based approach, or even a text-based approach, to discourse markers is inadequate, since the text alone is not the only source of relational signals. Multimodal signals could play a greater role in discourse interpretation than many current theories of discourse comprehension might lead us to expect. Future research focusing on signals in various modalities will inform a more comprehensive theory of constructing meaning in language.

A first avenue to explore is the signal∼meaning correlation between different types of relations and gestures. Can we build relation profiles for different gestures? And, in a similar vein, can we build gesture profiles for different connectives? Taking a bottom-up approach, corpus-based research can provide insight into which gestures typically co-occur with specific discourse relations. For example, one could imagine that a gesture consisting of fingers pinched together might co-occur with specification relations, which can be expressed lexically using “more specifically.” To be able to conduct such an investigation, a large amount of discourse-annotated video data are needed, since the signal∼meaning correlation might not be strong (that is, it is not clear how often a relation might be signaled using a specific gesture). Automatic labeling of connectives and discourse relations in transcripts of speeches, using a state-of-the-art discourse parser, might be a useful approach to gain more discourse-annotated data that can then be annotated for gestures.

A second avenue for future research is the interplay between explicit connectives and gestures. To isolate the effect of gestural discourse markers, we did not include any lexical discourse markers in the current study. It is still an open question to what extent gestures facilitate discourse processing in the presence of lexical markers. We expect an additive effect: in the presence of an ambiguous connective or other lexical signal (e.g., the connective *but* or syntactic parallelism as an underspecified signal for contrast relations), inferring the target relation would be facilitated in the presence of a gestural discourse marker, since comprehenders can exploit two congruent signals and thereby strengthen the support for their discourse expectations. There is also a range of research questions regarding how the comprehender deals with the combination of lexical and gestural discourse markers. For example, what happens when the lexical signal and the gesture do not match, such as when the speaker uses a contrastive gesture along with a causal connective?

Another question is how these two signals interact: When a relation is marked lexically, is the probability of the speaker providing a gesture higher or lower? Providing only one type of discourse relation signal, whether visual or lexical, might be more economical for both the speaker and the comprehender. Again, corpus-based studies could provide insight into the correspondence between connectives and gestures. Further, comprehension paradigms can provide insight into whether participants benefit from multimodal marking, or whether this might provide an information overload, in which case they revert to focusing only on a single modality.

A final avenue of interest is the possibility of individual differences in gesture comprehension. Research has shown that comprehension of connectives is associated with various potentially influencing factors at the individual level: variation in connective mastery is related to age (Nippold et al., 1992), academic background (Tskhovrebova et al., 2022a; Van Silfhout et al., 2015; Zufferey & Gygax, 2020), reading proficiency (Van Silfhout et al., 2015), linguistic experience (Scholman et al., 2024; Tskhovrebova et al., 2022a, 2022b; Zufferey & Gygax, 2020), and general reasoning skills (Scholman et al., 2024). In other words, people vary from each other in how well they know the meaning of connectives. Studies have shown similar findings for nonconnective signals: people differ from each other in how sensitive they are to quantifiers as signals for list relations (Scholman et al., 2020), and to prosody as a signal for subjectivity in causal relations (Hu et al., 2023).

Extending these findings to gestural signals, it is likely that not all comprehenders are equally capable of exploiting the information provided by gestural discourse markers. Indeed, participants in our study showed a high degree of variation, with the frequency of target continuations per participant ranging between 0% and 78%. It remains an open question what factors can explain these findings. Some participants might be less capable of simultaneously picking up on information from multiple modalities (i.e., information overload). Another possibility is that some participants have had less opportunities to learn specific signal∼meaning correlations (language exposure), or they might be less capable of learning those correlations (statistical learning skills). Future studies will hopefully provide more insight into the nature of individual differences in discourse gesture comprehension.

In sum, the current study investigated to what extent comprehenders can exploit the information provided by gestural discourse markers. Being able to establish discourse relations in communication is crucial to creating a mental representation of the message. Prior studies have focused on the effect of connectives, but many relations are, in fact, not marked by explicit connectives. It is likely that comprehenders rely on other signals to create their mental representation. However, little is known about what other signals people can use, especially signals in the spoken and visual modality. The results from the current study are the first to show that comprehenders can extract information from gestural discourse markers and use this to inform their discourse expectations. These results therefore emphasize the need for considering multimodal discourse signals in our theories of discourse comprehension.

## Data Availability

All materials, data, code and preregistration are available in an online repository: https://osf.io/68mcf/.

## Appendix. Written version of the experimental prompts

**Table:**

Item ID	Relation type	Prompt
02	List	Nathaniel has a crush on his classmate Suzy and wants to take her to prom. He told me he has an awesome proposal planned. He practiced a choreographed dance to an Ed Sheeran song.
03	List	Rose wanted to be an event planner, mainly focused on corporate events. She was explaining her two-year plan to start her business to her mother. She would become an intern at a well-known company.
06	List	John told me he was learning to cook. His mother’s birthday was coming up, and he wanted to surprise her with a home-cooked meal. He wanted to make a Waldorf salad for his mother.
08	List	Ruben is very concerned for the environment. At a family event, he was explaining to his uncle how the environment is in grave danger. The sea ice is melting at a shockingly rapid pace.
09	List	Yasmin was planning her birthday party with her best friend Cleo. They considered throwing the party together, since their birthdays were only a week apart. Yasmin wanted to buy a chocolate cake for the party.
10	List	Paul was known for being chaotic. He was already twenty minutes late for work and his wife asked him why he hadn’t left the house yet. Paul said that he could not find his car keys.
14	Contrast	Gillian had just moved into her new apartment. Since her fridge was empty, Gillian thought of asking her friend Mark to go shopping with her. She thought they could take the car to go shopping.
16	Contrast	Emily is mulling over what to do next, as she is unsatisfied with her job. She heard about the booming wool industry in New Zealand. She could perhaps move to New Zealand to farm sheep.
17	Contrast	Heather loves to travel solo, and has done so multiple times. She is considering what activities to do while on vacation in Hawaii next month. She really loves the idea of learning how to surf.
19	Contrast	Ronan is a very ambitious young man. He has been thinking about quitting his job as a supermarket clerk after working there for five years. He thinks he could get a promising job at a multinational.
20	Contrast	Melissa is a student living in California. Melissa’s new friends live at the seaside and now she is planning a weekend trip to visit them. She’d like to drive there straight after work on Friday.
22	Contrast	Lucy has been working as a well-paid web designer for the past ten years. Lucy is thinking about how best to manage her personal finances. She’d like to invest in crypto as soon as possible.
38	Exception	My friend Melissa has been a big Quentin Tarantino fan since she was 15. She has posters of his films plastered up on her walls. She has basically seen all Tarantino films that exist.
40	Exception	Alicia had to do chores around the house after school. Her mother asked her to mow the lawn, do the laundry, and clean the bathroom. Alicia had done the chores she was asked to do.
41	Exception	My friend Anna had two German Shepards, Billy and Zane, which she loved very much. They were big, loud, and full of love and fur. Every day she took them both on a forty minute walk.
44	Exception	Eric was very nervous and hopeful about his upcoming job interview, which was to take place next week at the company’s headquarters in southern California. Eric had never left his beloved home state of Minnesota.
46	Exception	Maggie wanted to buy a gift for her mother, since Mother’s Day was approaching. Maggie absolutely adored her mother, and they had a great relationship. Her mother had never been the biggest fan of flowers.
48	Exception	My friend Caroline told me she was taking yoga classes at the YMCA. She wanted to start a new, healthier hobby, and yoga seemed perfect. Caroline had not practiced sports for the past five years.
